# Glycerine as a Substitute for Cod-Liver Oil in Phthisis

**Published:** 1856-10

**Authors:** H. Wardner

**Affiliations:** Chicago, Ill.


*Glycerine as a substitute for Cod-liver Oil in Phthisis.* By H. Wardner, M.D., of Chicago, Ill.

In its internal use, glycerine has great advantage over cod-liver oil, on account of its blandness, and favoring instead of destroying the digestion, and nauseating the patient. In children, it is easy of administration, on account of its sweet and agreeable taste. In the latter part of October, 1855, by permission of Dr. N. S. Davis, I used it at the Mercy Hospital in the proportion of four parts to one of the syr. of the iodide of iron, in the case of a man about 40 years of age, who had been using the cod-liver oil, but was obliged to discontinue it, on account of its nauseating and irritating effects on the stomach. This man, at the time, was much emaciated, much troubled with coughing, and expectorating daily nearly a pint of thick, tenacious mucus, freely mixed with tubercular matter in a granular form. The infra-clavicular space was considerably fallen in, and all the symptoms and signs plainly evinced quite an advanced stage of the disease. He had been under treatment for several weeks, just previous, for chronic diarrhœa, which was not entirely cured. In about a week after commencing the use of this prescription, the cough and expectoration ceased, and the patient was much improved generally. He has continued its use up to the present time, Jan. 10, 1856' has regained his natural fullness and rotundity, and is fast regaining his strength.

Dr. Davis subsequently used the same remedy in a case of incipient tuberculosis, with equally satisfactory results. Dr. De Laskie Miller recently prescribed in a more advanced stage of the disease, with the most happy effects. In conclusion, I believe it a most valuable addition to the list of curative agents. —*N. W. Med. and Surg. Journ.*

